# Our New York Letter

**Published:** 1890-05

**Authors:** Wm. L. Russell

**Affiliations:** New York


### Correspondence.

### OUR NEW YORK LETTER.

NEW YORK, April 12, 1890.

When Koch announced nine years ago that he had discovered the essential element in the causation of pulmonary consumption, a new impetus was given to the study of that prevalent and fatal disease. Since then efforts have been principally directed toward investigating the characteristics of the bacillus, and the discovery of means to effect its destruction. Many varieties of treatment have been introduced with the latter end in view, from the inhalation of hot air and antiseptic vapors, to the internal administration of creosote and the rectal injection of sulphuretted hydrogen gas. Considerable attention has been paid to the subject in the societies here the past month, and the London and Paris letters of the *Medical Record* reveal that similar activity has been shown in the same direction in those places.

At the last meeting of the Practice of Medicine section of the Academy, "Pulmonary Comsumption in the Light of Modern Research" was the topic of discussion. The paper of the evening was read by Dr. S. S. Burt. He stated that while it was undoubtedly true there was no tuberculosis without the bacillus, it was equally true that previous impairment of the vitality of the tissues was an essential element. The hereditary nature of phthisis was long regarded as a settled fact. It was now known, however, that a structural weakness of certain organs, a deficient respiratory power and consequent impure blood were the conditions inherited. These could also be acquired. Hence it was that the disease was more prevalent among those who led sedentary lives. Lencocytes were thought to have some germicidel power, or to be at least he scavengers of the system, and fresh blood serum was certainly destructive to bacteria. Hence it was that impoverished blood formed fruitful soil for the growth of the tubercular bacilli. Besides impairing the quality of the blood, sedentary occupation exposed those engaged in it to many sources of contagion. Tuberculosis had been produced in dogs by inoculating them with the dust of floors and with scrapings from walls, and a similar effect was produced by the inhalation of dried sputa containing the bacillus. It was quite common to find at autopsies traces of recovered lung disease, and it seemed as though tuberculosis was inclined to self-limitation. Perhaps then if all cases were known the death rate would not seem so alarming.

When the question of treatment was approached it was necessary to keep in view the predisposing and exciting causes. The carelessness of mankind regarding the physical character of their offspring was accountable for much suffering. A man with a tendency to phthisis ought to understand that he became a potent source of evil if he married a woman with a similar tendency. If but one parent was of consumptive tendency, however, the children might grow up to healthy life if intelligently reared. Such children should spend most of their time out of doors, and be protected from enfeebling influences at home and at school.

No drug had yet been discovered which could be said to cure phthisis. Elevation of the tone of the tissues and fluids of the body to a point where bacteria did not thrive in them was the principal object to be aimed at. Migration promised much, but there was nothing specific in any climate. Some improved in a warm and others in a cold climate, and it was impossible to tell beforehand which would answer best. A sedative mild atmosphere for the feeble, nervous patient, and stimulating mountain air for the more robust, were good general rules to follow. One should not be too hasty either in sending patients from home, for the most satisfactory recovery that he had ever seen occurred in a poor dispensary patient right here in New York.

In the discussion which followed, Dr. A. Jacobi spoke of the rare occurrence of tuberculosis by direct transmission from parent to child. But one case had come under his observation, and in this the child was still-born. Children of tuberculous parents, however, were often found to have small hearts, or stenosis or congenital smallness of the pulmonary artery. Another important point was the condition of the epithelium, which was often abnormal. This was interesting in view of the fact that the normal cylindrical and fimbriated epithelium of the air passages prevented injurious substances from penetrating them.

Dr. Wm. H. Draper said that it was a sad fact that the discovery of Koch had done very little to mitigate the evils arising from consumption. Indeed it had in some measure but complicated the problem, for the role of the bacillus was not yet fully understood. It was found in lupus and yet there was no infection, and it frequently remained for a long time in the lymphatic glands without causing any mischief whatever. It was now so widely distributed that we were helpless in preventing infection; it being impossible to wage a war of extermination. He knew of no hospital in New York, at least, where the valuable suggestions made regarding the disinfection of sputa were carried out. So far as the practical management of the disease was concerned, the outlook was extremely discouraging.

Dr. A. H. Smith believed that the impetus given to the search for specifics by the discovery of the bacillus was leading us away from other important matters in the management of phthisis. Some method of preventing the propagation of the bacillus would yet be found, but all we could do in this direction at present was to warn the patient of the danger of contagion. Among the rural population the disease was often conveyed from one member of a family to another till all but the parents were swept away. This was because of the small sleeping-rooms with low ceilings, which were occupied by more than one person, fresh air being in the meantime most carefully excluded. The relation of bronchial catarrh to tuberculosis was a matter of much interest. He believed that it was frequently the starting-point of the disease, perhaps because the diseased epithelium was unable to prevent the entrance of the bacilli. In regard to treatment, he had seen remarkable results from supplementing the ordinary methods of feeding with rectal alimentation. He be–

lieved that the future would show this to be an extremely valuable measure.

Dr. R. C. M. Page was of the opinion that statistics showing that tuberculosis was a self-limited disease were wanting. He believed that marriages between consumptives should be prohibited.

Dr. J. W. Roosevelt remarked that though the bacterial origin of phthisis had been proven, it was certain that other conditions were requisite. The light which modern science had thrown on the subject was as yet very faint. Under present conditions, however, it was our duty to destroy the bacillus as far as possible. To attempt this by means of antiseptic medication was, however, irrational. The hot air treatment was certainly useless. The bacilli were surrounded by albumen, which would be coagulated by the degree of heat required to destroy them. He did not think that it had been satisfactorily explained yet why the upper lobes were usually attacked first. He had noticed that when tuberculosis supervened on chronic bronchitis and emphysema it was usually diffused. This might perhaps be due to the diseased condition of the bronchial mucous membrane, allowing a more general inoculation.

Dr. Brush stated that so far as he had been able to learn, in every case of direct transmission of tuberculosis from mother to offspring there had been abortion, or the child had been still-born. In relation to this matter he had made an experiment. He had shut up several hens and a cock, and for ten days had fed them on the lungs of a cow which had died of tuberculosis. At the end of the period one of the hens was killed and unmistakable evidence of tuberculosis was discovered. Twenty-six eggs had been laid by the confined hens, and these were now placed under other hens. At the end of the usual period the hens broke the shells, and it was found that twenty-five chicks had developed but not one was alive.

At the last meeting of the Academy Dr. McG. Thompson gave an account of a series of experiments made on animals, which revealed the fallacy of the hot-air treatment. He had found that after a dog had inhaled dry air at a temperature of from 200° to 300° F., for an hour, the temperature of his lungs was not at all elevated, and after the inhalation had been continued still longer it was raised only two or four degrees, and the tracheal temperature four or six. In commenting on these experiments, Dr. J. Lewis Smith stated that about two years ago, one of the Weigert brothers, while visiting this city, had tried the hot-air treatment on the consumptives in Charity Hospital. The results were not so good as those now obtained by the use of antiseptic inhalations, such as a mixture of ten parts of terebene and two of creosote, the latter being given internally at the same time. This plan of treatment had superseded that by cod-liver oil in this hospital.

In the Paris letter of the *Medical Record* of March 29, it is stated that at the last meeting of the Academy of Medicine of Paris, Dr. Dujardin Beaumetz expressed the opinion that in view of the non-success of the specific treatment of phthisis, we should aim at the amelioration of the hygiene of the patient. Proper aeration would be real progress, but rational pharmaceutical treatment should be added. These remarks were called forth by a paper by Dr. Nicaise in which the matter of aeration was especially insisted on. It was stated that at the establishment of Dr. Dettneoler at Falkenstein, where the patients were kept out of doors in all seasons, twenty-five per cent. of absolute and twenty-seven per cent. of relative cures were obtained. Dr. Nicaise's researches showed that a room with a southern exposure and a chimney fire could be kept at a temperature of 50° F. at night, when the external temperature was as low as 35.6° F. and the window open a foot or sixteen inches, the shutters being closed.

At the last meeting of the County Society of New York, Dr. J. A. Andrews stated that he had found the micro-organism of gonorrhœa, the genococcus, in seventy-two out of seventy-three cases of purulent ophthalmia in adults, and in every one of a hundred and three cases of the same disease in the new-born. Out of nine cases among infants two or three months old, he had found the micro-organism in three. Dr. David Webster, of the Manhattan Eye and Ear Hospital, also stated that the genococcus had been found in the pus which he had caused to be examined from a large number of cases occurring in infants. Almost nothing new was suggested in the way of treatment. Dr. H. J. Hepburn remarked, however, that at the Randall Island Hospital for Children a solution of bichloride of mercury, one to twenty thousand, was now used for cleansing, and that since the introduction of this plan of treatment it had not been found necessary to protect the sound eye, as not a single case of contagion to it or to the nurse's eye had occurred. If perforation of the cornea occurred it was customary to revert to the usual saturated solution of boracic acid. WM. L. RUSSELL.

